# Effects of electrospun fibers containing ascorbic acid on oxidative stress reduction for cardiac tissue engineering

**DOI:** 10.1002/app.54242

**Published:** 2023-05-26

**Authors:** Ella‐Louise Handley, Anthony Callanan

**Affiliations:** ^1^ Institute for Bioengineering, School of Engineering University of Edinburgh Edinburgh UK

**Keywords:** antioxidant scaffold, ascorbic acid, cardiac tissue engineering, electrospinning, myocardial infarction

## Abstract

Tissue engineering provides promise for regeneration of cardiac tissue following myocardial infarction. However, the harsh microenvironment of the infarct hampers the efficacy of regenerative therapies. Ischemia‐reperfusion injury dramatically increases the levels of reactive oxygen species (ROS) within the infarcted area, causing a cascade of further cellular injury. Implantable tissue engineered grafts can target this oxidative stress by delivering pharmaceutical compounds directly into the diseased tissue. Herein, we successfully fabricated electrospun polycaprolactone (PCL) fibers containing varying concentrations of ascorbic acid, a potent antioxidant well known for its ROS‐scavenging capabilities. The antioxidant scaffolds displayed significantly improved scavenging of DPPH radicals, superoxide anions and hydroxyl radicals, in a dose dependent manner. Mechanical properties testing indicated that incorporation of ascorbic acid enhanced the strength and Young's modulus of the material, correlating with a moderate but non‐significant increase in the crystallinity. Moreover, the scaffolds supported adhesion and maintained survival of human umbilical vein endothelial cells in vitro, indicating good cytocompatibility. These results provide motivation for the use of ascorbic acid‐containing fibrous scaffolds to regulate the highly oxidative microenvironment following myocardial infarction.

## Graphical abstract

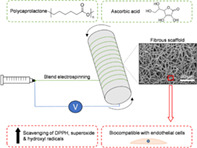

## 1 INTRODUCTION

Cardiovascular diseases (CVDs) remain the leading cause of death worldwide; it is estimated that over 9 million people die from ischemic heart diseases globally, accounting for 16% of all deaths.^1^ The blockage of a major coronary artery results in myocardial infarction, characterized by a loss of large numbers of cardiomyocytes.^2^ Ischemia‐reperfusion injury, mitochondrial dysfunction, and the acute inflammatory response caused by myocardial infarction leads to the production and accumulation of ROS.^3^ The subsequent oxidative stress causes a cascade of myocardial injury; cells undergo apoptosis due to DNA damage and protein oxidation, and the infiltration of M1 macrophages and production of pro‐inflammatory cytokines occurs.^4^ Regulation of this microenvironment toward a healthier state may increase the efficacy of current regenerative therapies and directly improve cardiac function.^5^ One treatment pathway is using exogenous antioxidants such as ascorbic acid (AA), α‐tocopherol and *N*‐acetylcysteine, which reduce overall oxidative stress via the direct scavenging of ROS or inhibiting their generation.^6^

Antioxidant therapies following myocardial infarction have been reviewed extensively^3^, ^6^, ^7^, ^8^, ^9^, ^10^; while preclinical studies administering exogenous antioxidants have reported reductions in infarct size and improvements in cardiac function, clinical trials have yielded limited success. It has been suggested that a major issue impacting clinical translation is the route of administration and attaining the therapeutic window within the diseased tissue.^6^, ^9^ Clinical trials to date have only administered antioxidants orally or intravenously, however targeting oxidative stress directly within the diseased site using implantable or injectable materials may more effectively reduce local oxidative stress.^11^ As such, considerable progress has been made in the development of microenvironment modulating biomaterials for cardiac applications, with a major focus on antioxidant materials that can scavenge ROS within diseased sites.^12^, ^13^, ^14^, ^15^, ^16^, ^17^, ^18^, ^19^, ^20^, ^21^

AA, commonly known as vitamin C, is a non‐toxic antioxidant that has been researched extensively for the treatment of myocardial infarction.^22^, ^23^, ^24^ A recent systematic review indicated that AA may decrease oxidative stress and inflammation, thus preserve cardiac function, when administered prior to reperfusion.^23^ Alongside direct implantation into the infarct, materials provide the advantage of sustained and tailorable release rates of the loaded pharmaceutical compounds.^25^ Recently, Guo et al. developed a hydrogel system conjugating AA to chitosan, with improved antioxidant properties and cardioprotective effects.^26^ However, synthetic polymers such as PCL display superior mechanical properties compared with natural materials such as chitosan.^27^ Additionally, AA and its oxidized forms are highly unstable in aqueous solution, especially under physiological conditions.^28^ Due to its hydrophobic nature, PCL may protect the vitamin from degradation while providing a sustained release, improving and extending the graft's antioxidant capabilities.^13^, ^29^

Tissue engineering approaches can be utilized as novel delivery mechanisms.^30^ Electrospinning offers the advantage of straightforward manipulation of scaffold morphology to mimic the architecture of the native tissue.^31^ Electrospun fibers have previously encapsulated a range of bioactive molecules such as proteins,^32^, ^33^, ^34^ decellularized extracellular matrix (ECM),^35^, ^36^, ^37^ and various molecules with antioxidant properties.^20^, ^38^, ^39^, ^40^, ^41^, ^42^, ^43^ AA is known to stimulate growth of and production of collagen by fibroblasts,^44^ thus has been previously encapsulated within PCL/hyaluronic acid blended fibers^45^ and polylactic acid/PCL copolymer fibers^46^ for skin tissue engineering, and polylactic acid fibers to promote ECM production for pelvic floor repair.^47^ Within cardiac tissue engineering, coaxial electrospinning has been used to develop PCL and gelatin nanofibers incorporating both an AA derivative and salvianolic acid B, a Chinese herbal medicine.^39^ However, blended electrospun fibers reportedly exhibit a more sustained release of AA compared with coaxial electrospun fibers.^25^

To the best of our knowledge, fibrous PCL and AA blended scaffolds have not been investigated in vitro using human vascular endothelial cells. Thus, in this study we developed electrospun PCL fibers containing AA and characterized the fibers using scanning electron microscopy (SEM) imaging, tensile testing, differential scanning calorimetry, water contact angle, and a range of radical scavenging assays. Additionally, we performed in vitro biocompatibility analyses using human umbilical vein endothelial cells (HUVECs).

## 2 MATERIALS AND METHODS

### 2.1 Materials

For scaffold fabrication and characterization, l‐AA (99%), PCL (average *M*_n_ = 80,000 Da), 2,2‐diphenyl‐1‐picrylhydrazyl (DPPH), hydrogen peroxide (30% wt/wt in H_2_O) (H_2_O_2_), Ampliflu™ Red (≥98.0%), peroxidase from horseradish (~150 U/mg) and pyrogallol (ACS reagent) were purchased from Sigma‐Aldrich, United Kingdom. Hexafluoroisopropanol (HFIP) (97%) was purchased from Manchester Organics, United Kingdom. Methanol (Analytical Research Grade) was obtained from ThermoFisher Scientific, United Kingdom; dimethyl sulfoxide (DMSO) (>99.5%) was obtained from Fisher Scientific, France; and Eagle's minimum essential media (MEM) was obtained from Gibco™, ThermoFisher Scientific, United Kingdom.

For in vitro work, HUVECs isolated from the umbilical cord of an infant Caucasian male were purchased from Promocell GmbH, Germany. MCDB131 Medium, Penicillin–Streptomycin (10,000 U/mL) and l‐glutamine were all acquired from Gibco™, ThermoFisher Scientific, United Kingdom. Fibroblast growth factor, vascular endothelial growth factor, epidermal growth factor, and insulin‐like growth factor were purchased from Peprotech, United Kingdom. Trypsin–EDTA solution (0.5 g porcine trypsin, 0.2 g EDTA), hydrocortisone, papain, cystein HCL, EDTA and DNA free water were obtained from Sigma‐Aldrich, UK. Finally, the Quant‐iT™ PicoGreen™ dsDNA Assay Kit and fetal bovine serum were acquired from ThermoFisher Scientific, United Kingdom, while the CellTiter‐Blue® Cell Viability Assay was supplied by Promega, United Kingdom.

### 2.2 Preparation of the electrospun scaffolds

l‐AA was homogenized in HFIP using a Dounce tissue homogenizer to give final concentrations of 0.15 wt/vol% and 0.3 wt/vol%. 10 wt/vol% PCL was added into the solution; the control scaffold was 10 wt/vol% PCL only. Solutions were placed on a tube roller at room temperature overnight. An EC‐DIG electrospinner (Serial no. 140450202, IME technologies, Netherlands) was used with software version 1.91. Antioxidant scaffolds are referred to via their respective AA content in the polymer solution: 0.15% AA and 0.3% AA.

**FIGURE 1:**
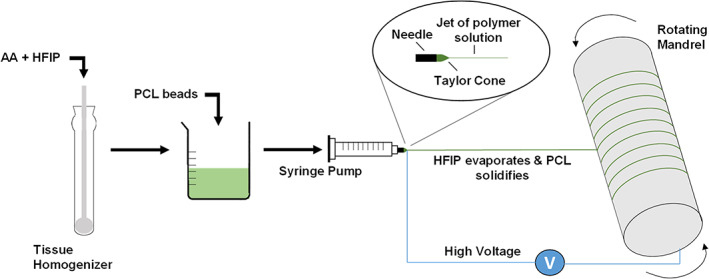
Schematic of the electrospinning process. [Color figure can be viewed at wileyonlinelibrary.com]

Solutions were loaded into 10 mL syringes and placed in a syringe pump (Harvard Apparatus, United States). Initial electrospinning parameters were decided based on Phillips et al.^44^ with the voltages and flow rate adjusted during the process until a stable Taylor cone was observed. The methodology was further optimized to produce the desired scaffold morphology with uniform fiber diameters across the mat. Final electrospinning parameters were kept constant for each scaffold and are shown in Table 1. A positive voltage of 14 kV was applied to the needle and fibers were collected on a rotating mandrel with an applied negative voltage of −4 kV. Scaffolds were left in a fume hood overnight to allow for evaporation of excess solvent, then stored at 4°C while protected from light to minimize oxidation of the AA. A schematic of the process can be seen in Figure 1.

**TABLE 1:** Parameters used in the electrospinning process.

Parameter	Value
Needle diameter	0.3 mm
Distance between needle tip and mandrel	120 mm
Flow rate	1 mL/h
Total volume of solution	6 mL
Applied voltages	+14, −4 kV
Mandrel rotational speed	250 RPM

### 2.3 Scaffold sterilization

Scaffolds were punched using a circular biopsy punch and placed into a suspension well‐plate. Scaffolds were sterilized by immersing in 70% ethanol for 10 min, then washed three times in 1 mL of phosphate buffered saline (PBS) for 10 min each.

### 2.4 Scaffold characterization

#### 2.4.1 Scaffold morphology

Ten millimeter scaffolds were punched using a circular biopsy punch and imaged using a TM4000 tabletop SEM (Hitachi) with a 10 kV accelerating voltage and backscattered electron detection mode. Images were uploaded to ImageJ and fiber diameters analyzed by computing the number of pixels per unit length using the image scale bar then superimposing straight lines across the fibers.

#### 2.4.2 Tensile testing

The mechanical properties of the scaffolds were measured as previously described^48^, ^49^, ^50^ using an Instron 3367 tensile testing machine (Instron) with a 50 N load cell. Rectangular samples of 10 mm by 40 mm were cut out and tested until failure using a strain rate of 50% per minute, a starting gauge length of 20 mm and preload of 0.01 N.

The thickness of each sample was measured using digital calipers. Incremental Young's modulus was calculated using previous methodology^51^, ^52^ between strain bands 0%–5%, 5%–10%, 10%–15%, and 15%–20%.

#### 2.4.3 Differential scanning calorimetry

The crystallinity of the scaffolds was analyzed using a DSC 8000 (PerkinElmer). Polymer properties are affected by both their thermal history and their morphology, thus ISO standards recommend heating and cooling runs be carried out twice.^53^ The first run reflects the polymer properties “as received.” The sample is then melted and cooled at a specified rate, erasing the influence of thermal and processing history on the properties.

Samples of 5 mg weight were cut and subjected to two thermal cycles between 10 and 75°C at a heating and cooling rate of 10°C/min. Measurements were carried out under a nitrogen atmosphere, using a nitrogen flow rate of 20 mL/min. The sample was held for 1 min at 75°C on the first cycle to erase thermal history. Results of both heating cycles are presented.

The degree of crystallinity, *X*_c_, was calculated using:

(1) $$X_{c}\left(\%\right)=\frac{\Delta H_{m}}{\Delta H_{0}}\times 100$$

Where Δ*H*_m_ is the enthalpy of fusion and Δ*H*_0_ is the enthalpy of fusion of an 100% crystalline sample (139.5 J/g for PCL^54^, ^55^).

#### 2.4.4 Wettability

Water contact angle measurements were undertaken using the sessile drop method. Briefly, a 5 μL droplet of diH_2_O was placed onto dry scaffolds, while an image was captured every 0.2 s using a DMK 41 AU02 monochrome camera. Analysis was undertaken using the Contact Angle plugin on ImageJ.

### 2.5 Antioxidant activity measurements

#### 2.5.1 DPPH assay

Twenty millimeter circular scaffold discs were punched and incubated for 30 min at room temperature with 1 mL of a 500 μM solution of DPPH in methanol, while being protected from light. Oxidation of the DPPH radicals induces a color change from magenta to yellow, therefore the percentage reduction can be quantified by measuring absorbance at 517 nm. A Clariostar® Plus Microplate Reader (BMG Labtech) was used, and the following equation employed:

(2) $$\begin{array}{c} \text{Percentage scavenging of DPPH radicals}=\left(A_{B}-\frac{A_{S}}{A_{B}}\right)\times 100 \end{array}$$

Where *A*_B_ and *A*_S_ correspond to the absorbance of the blank and the absorbance of the sample, respectively.

#### 2.5.2 Hydrogen peroxide assay

Twenty millimeter scaffolds were punched and plated in a 12‐well suspension plate, then sterilized according to Section 2.3. One hundred micromolar of H_2_O_2_ in MEM was prepared and 1 mL added to each scaffold. The plate was incubated at 37°C while protected from light and media samples were taken at 1, 3, and 6 days.

The concentration of H_2_O_2_ in the samples was measured using the Amplex Red/Horseradish Peroxidase method, as previously described.^56^ Briefly, a master mix was prepared by adding 50 μL of 10 mM Ampliflu™ Red in DMSO and 100 μL of 10 U/mL lyophilized horseradish peroxidase in PBS, to 4.85 mL of PBS. The peroxidase reacts with H_2_O_2_, producing oxidants that convert the Ampliflu™ Red into resorufin, a fluorescent compound that can be measured quantitatively.

Our previous results indicated the detection limit of the assay to be 25–30 μM, thus samples were diluted 1:4 using PBS to lower the H_2_O_2_ concentration into the detectable region. 50 μL of the diluted sample was incubated with 50 μL of the master mix at room temperature for 30 min while protected from light. A Clariostar® Plus Microplate Reader was used to measure the fluorescence at excitation 540–15 nm and emission 590–20 nm, using a 563.8 nm dichroic mirror. A set of standard solutions were prepared by diluting H_2_O_2_ in PBS.

#### 2.5.3 Superoxide assay

Twenty millimeter scaffolds were punched and incubated with 1 mL of 3 mM pyrogallol in Tris–HCL (pH = 8.1) at room temperature while protected from light. In alkaline solution pyrogallol auto‐oxidizes, producing superoxide anions (O_2_^•–^). The scavenging of the anion can be determined by measuring the absorbance of the reaction product purpurogallin.^57^ After 5 min, a 100 μL sample was taken into a 96‐microwell plate and absorbance measured using a Clariostar® Plus Microplate Reader at 320 nm.

### 2.6 Cell studies

#### 2.6.1 HUVEC cell culture and seeding

HUVECs were expanded using our previous methodology.^37^ Briefly, HUVECs were cultured using MCDB131 medium supplemented with 5% vol/vol fetal bovine serum, 1% l‐glutamine, 1% penicillin/streptomycin, 1 mg/L hydrocortisone, 2 μg/L fibroblast growth factor, 1 μg/L vascular endothelial growth factor, 10 μg/L epidermal growth factor, 2 μg/L insulin‐like growth factor, and 50 mg/L AA. At 80% confluency, cells were lifted using trypsin–EDTA solution, centrifuged at 220 RCF for 5 min and the supernatant discarded. Cells were resuspended in media and counted using 0.4% trypan blue and a hemocytometer.

Ten millimeter diameter circular scaffolds were sterilized according to Section 2.3, then left to soak overnight in 1 mL of supplemented media (37°C, 5% CO_2_). HUVECs at passage 7 were seeded onto scaffolds at a density of 40,000 cells/cm^2^. Briefly, 50 μL of cells suspended in complete medium were dripped onto scaffolds and incubated (37°C, 5% CO_2_) for 2 h to allow for attachment, before an extra 1 mL of media was added. For scaffold culture, AA was emitted from the culture medium. The medium was changed at 24 h and timepoints were taken on days 1, 3, and 6 following media change. Cells were cultured on tissue culture plastic (TCP) as a negative control.

#### 2.6.2 Cell viability assay

Cell viability was evaluated using a CellTiter‐Blue® (CTB) assay kit. The CTB solution contains resazurin, a non‐fluorescent dye that is converted into resorufin by metabolically active cells.^58^ Resorufin is highly fluorescent and, in most cases, the fluorescent signal is proportional to the cell viability.

Briefly, a mixture of 1:5 CTB: complete media was prepared. Supernatant was aspirated and replaced with 1 mL of the CTB solution. Plates were protected from light and incubated (37°C, 5% CO_2_) for 4 h. A 100 μL sample was placed into a black microwell plate and fluorescence was measured at 560 nm excitation and 590 nm emission using a Clariostar® Plus Microplate Reader.

#### 2.6.3 DNA quantification

Scaffolds were washed three times with PBS, stored at −80°C and then lyophilized overnight. A solution of 1.5 units papain, 5 mM cysteine HCl and 5 mM EDTA in DNA free water was prepared and added to the samples. Scaffolds were subsequently dissolved by incubating them overnight at 65° and vortexed periodically.

The Quant‐iT™ PicoGreen double‐stranded DNA (dsDNA) assay kit was performed according to the manufacturer protocols.^59^ The assay contains a fluorescent stain that binds specifically to dsDNA. Briefly, scaffolds were incubated for 5 min at room temperature with the PicoGreen working solution; a 100 μL sample was transferred to a black multiwell plate and the fluorescence was measured at 480 nm excitation and 520 nm emission using a Clariostar® Plus Microplate Reader.

### 2.7 Statistical analysis

Statistical analysis was undertaken with Origin 2021bl software using a one‐way analysis of variance test with Tukey's test for post hoc analysis. Results are displayed as mean ± SD; error bars display SD. Unless otherwise specified, * indicates *p* ≤ 0.05, ** indicates *p* ≤ 0.01, and *** indicates *p* ≤ 0.001.

## 3 RESULTS

### 3.1 Scaffold characterization

**FIGURE 2:**
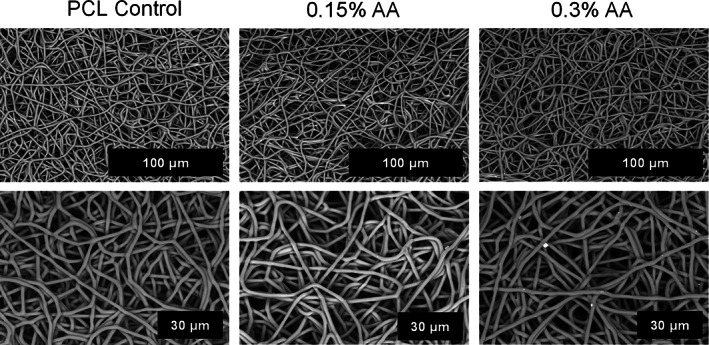
Representative SEM images of the three scaffolds at 1500× and 3000× magnification.

Three fibrous mats were successfully fabricated via electrospinning. The SEM images in Figure 2 display their similar morphologies: fibers are continuous, randomly aligned and appear to be smooth. The fiber diameters were consistent between groups, ranging from 1.81 to 1.88 μm.

All scaffold groups were hydrophobic (*θ* > 90°) (Table 2), where an increase in the AA content correlated with a slight but non‐significant increase in the water contact angle.

**TABLE 2:** Mean scaffold fiber diameters taken from ImageJ analysis (*N* = 50), contact angles measured by the Sessile drop method, and ultimate tensile strength measured via tensile testing (*N* = 4).

	Fiber diameter (μm)	Contact angle at 1 s (°)	Ultimate tensile strength (MPa)
PCL Control	1.82 ± 0.14	127.8 ± 3.5	2.72 ± 0.42** ^,^ ***
0.15% AA	1.81 ± 0.15*	125.1 ± 1.6	3.74 ± 0.30**
0.3% AA	1.88 ± 0.14*	123.4 ± 1.2	4.20 ± 0.22***

* *p* ≤ 0.05;

** *p* ≤ 0.01;

*** *p* ≤ 0.001.

**FIGURE 3:**
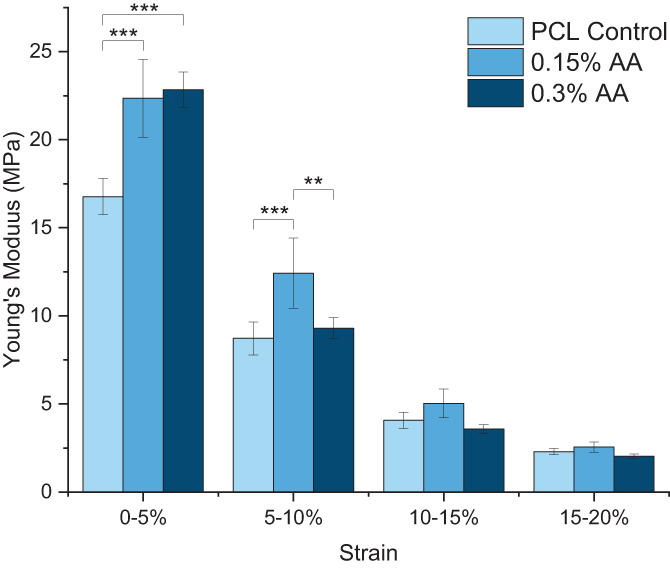
Incremental Young's modulus of each scaffold at different strain bands (*N* = 4) **p ≤ 0.01; ***p ≤ 0.001. [Color figure can be viewed at wileyonlinelibrary.com]

Tensile properties were determined using an Instron tensile tester (Figure 3, Table 2). Differences were noted in the Young's modulus within groups at each strain band, with the stiffness decreasing in every group with increasing strain. Between the 0%–5% strain, both antioxidant scaffolds were significantly stiffer than the PCL control, while at 5%–10% strain the 0.15% PCL AA scaffold exhibited a significantly greater Young's modulus than both the PCL control and the 0.3% AA scaffold. Within the 10%–15% and 15%–20% strain bands, no differences were noted between the scaffold groups. Finally, incorporation of AA influenced the strength of the scaffold, with the 0.15% and 0.3% AA scaffolds displaying a 1.38× and 1.5× significant increase in the ultimate tensile strength, respectively, compared with the PCL control.

**FIGURE 4:**
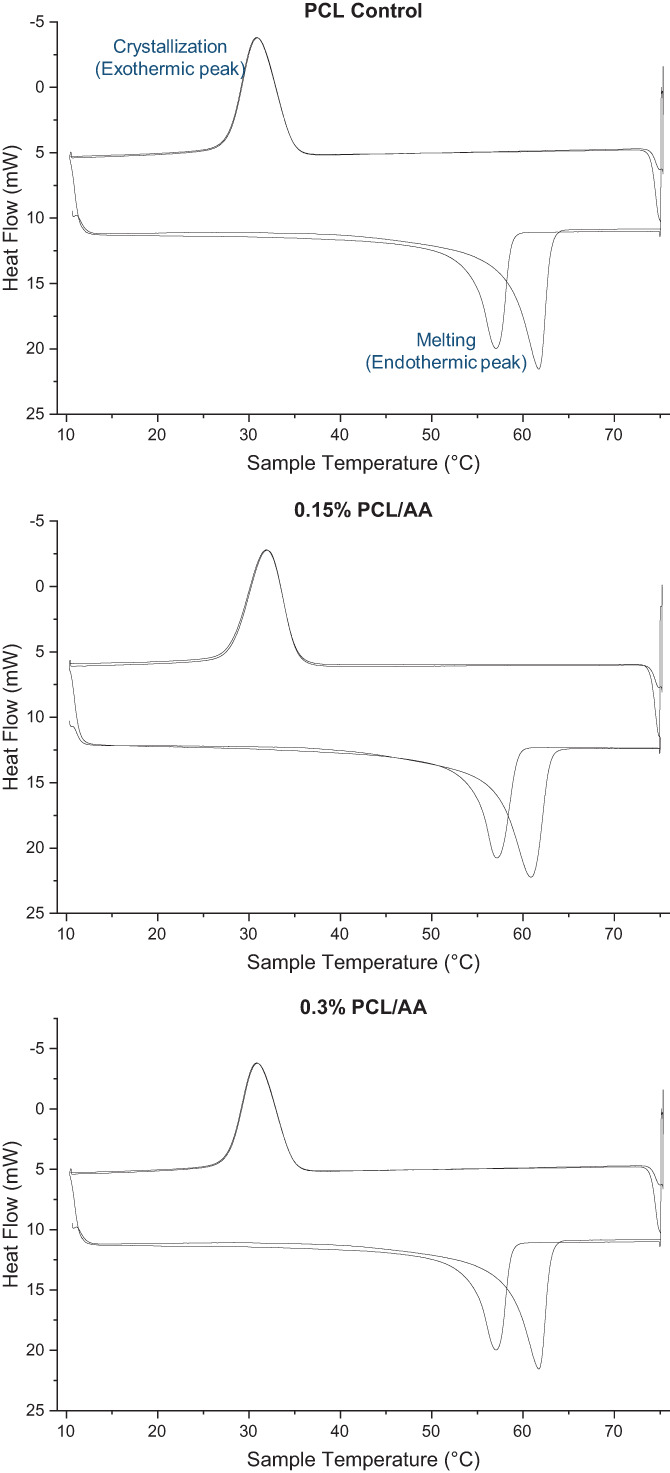
Representative DSC plots of each sample. [Color figure can be viewed at wileyonlinelibrary.com]

Thermal properties were evaluated both “as received” and after common thermal treatment (Table 3). Figure 4 shows representative differential scanning calorimetry (DSC) plots of heat flow against sample temperature for each scaffold. The plots are indicative of a semi‐crystalline polymer,^60^ with the endothermic and exothermic peaks representing the melting and cold crystallization points, respectively. Incorporation of AA into the fibers increased the crystallinity, though no significance was observed between groups. Similarly, no differences were detected between the melting temperatures (*T*_m_) of the scaffolds during the first thermal cycle. Melting points and degree of crystallinity decreased within all groups after annealing.

**TABLE 3:** Scaffold thermal properties measured using differential scanning calorimetry (*N* = 4).

	First thermal cycle			Second thermal cycle	
	*X*_c_ (%)	*T*_m_ (°C)	*T*_c_ (°C)	*X*_c_ (%)	*T*_m_ (°C)
PCL Control	46.3 ± 2.7	61.6 ± 0.4	30.6 ± 0.3**	35.9 ± 2.3	57.4 ± 0.5
0.15% AA	52.3 ± 11.1	61.8 ± 0.4	30.4 ± 0.2^†^	41.8 ± 7.8	57.7 ± 0.2*
0.3% AA	50.3 ± 2.0	61.2 ± 0.7	31.6 ± 0.5** ^,^ ^†^	37.6 ± 2.2	57.0 ± 0.2*

Abbreviations: *T*_c_, crystallization temperature; *T*_m_, melting point.

* *p* ≤ 0.05;

** *p* ≤ 0.01;

^†^ *p* ≤ 0.01.

### 3.2 Intrinsic antioxidant activity

**FIGURE 5:**
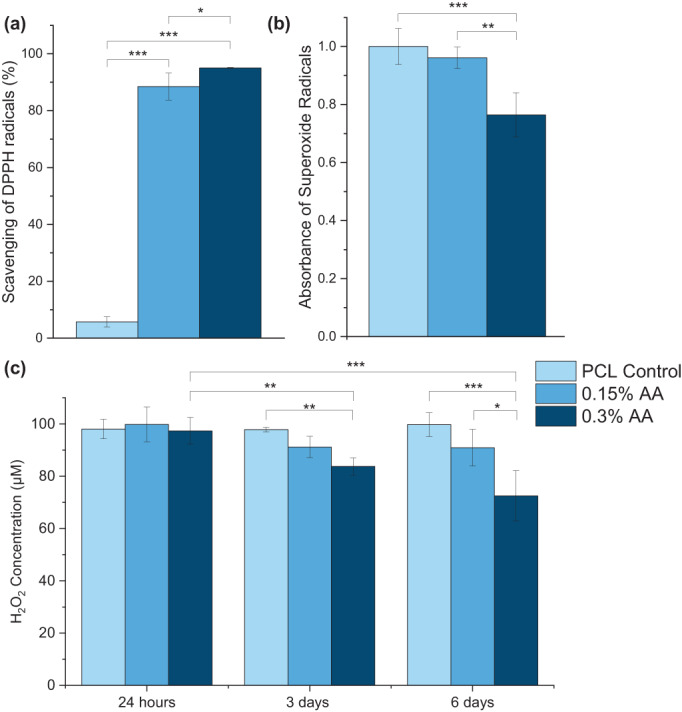
Scavenging of (a) DPPH radicals and (b) superoxide anions, (c) change in H_2_O_2_ concentration following incubation with scaffolds (normalized to account for degradation of H_2_O_2_ in media). (*N* = 4), *p ≤ 0.05; **p ≤ 0.01; ***p ≤ 0.001. [Color figure can be viewed at wileyonlinelibrary.com]

When incubated with a DPPH/MeOH solution, the antioxidant scaffolds scavenged over 90% of the DPPH radicals, while the PCL control scavenged less than 6% (Figure 5a). Interestingly, no differences were seen between the two antioxidant scaffolds. Lower inhibition was seen with the superoxide radicals compared with the DPPH radicals (Figure 5b). Although greater scavenging was observed by both the antioxidant scaffolds than the control, significance was only observed with the 0.3% AA scaffold.

The concentration of H_2_O_2_ within media decreased significantly when incubated at 37°C, therefore results of the H_2_O_2_ assay are presented normalized to the control group (Figure 5c). As expected, no change was detected within the PCL only group. Interestingly, only the 0.3% AA scaffold group displayed significant antioxidant capacity against H_2_O_2_, reducing the concentration by 16% and 27% after 3 and 6 days, respectively. Comparatively, the 0.15% AA scaffold scavenged 9% of H_2_O_2_ by day 3, though no significance was observed, and no further reduction was seen at day 6.

### 3.3 In vitro analysis

**FIGURE 6:**
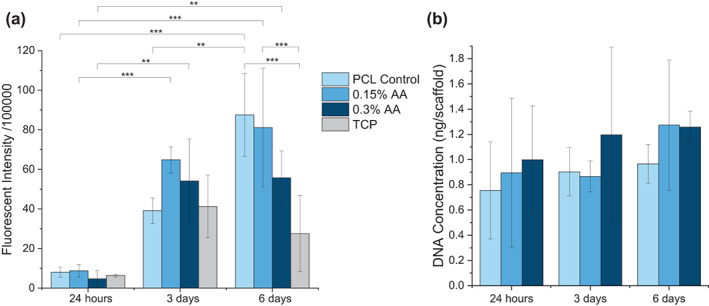
(a) Viability of human umbilical vein endothelial cells (HUVECs) cultured on all scaffolds for up to 6 days, measured using the CellTiter Blue Assay, (b) dsDNA quantification using PicoGreen Assay for all scaffolds. (*N* = 4), **p ≤ 0.01; ***p ≤ 0.001. [Color figure can be viewed at wileyonlinelibrary.com]

The biocompatibility analyses indicate cell survival for up to 6 days within all groups. Viability was determined using the CellTiter‐Blue® Assay; fluorescence increased significantly between timepoints within all groups (Figure 6a). On day 6, the PCL control, 0.15% AA and 0.3% AA scaffolds each exhibited 3.1×, 3.0×, and 2.0× greater viability, respectively, compared with the TCP control, indicating the cells may be capable of proliferating to a greater extent on the scaffolds. Additionally, the dsDNA results (Figure 6b) show that all scaffold groups contained DNA over the course of 6 days, though no significant difference was observed between time points or groups.

## 4 DISCUSSION

All cells possess a range of endogenous protective mechanisms that reduce or prevent injury due to external stressors. For example, exposure to elevated temperatures activates the heat‐shock response, whereby molecular chaperones detect unfolded proteins and either facilitate their refolding or their destruction.^61^ The hypoxia inducible factors are a set of transcription factors that serve to regulate homeostasis in response to insufficient levels of oxygen^62^; similarly, a number of enzymes including superoxide dismutase, catalase and glutathione peroxidase regulate intracellular ROS.^10^ Usually these stress responses work effectively to maintain homeostasis and proper functioning of cells and tissues. However, during myocardial reperfusion the sudden influx of oxygen generates excessive ROS, overwhelming the tissue's inherent antioxidant capabilities.^10^ ROS inhibit the bioavailability of nitric oxide, an important signaling molecule with significant cardioprotective qualities, modulate the inflammatory response, and cause direct damage to enzymes, membrane lipids and DNA.^63^ Despite promising results in vivo, regenerative therapies for myocardial infarction have shown limited success in clinical trials. Research suggests that modulating the infarcted microenvironment toward a less diseased state may enhance the efficacy of these interventions.^5^

It has been established that high doses of AA (reported as greater than 6 g per day, or 75 mg per kg per day), are safe in human populations.^64^ Clinical trials have suggested infusing AA prior to reperfusion improves cardiac function,^22^, ^23^ however there are issues in traditional antioxidant therapy regarding obtaining therapeutic concentrations within the diseased tissue.^6^ Direct delivery of antioxidants into the area of interest using a biomaterial can increase their local bioavailability.^65^ PCL, an FDA approved biocompatible polymer, has been previously utilized as a delivery system for a range of antioxidant molecules including α‐tocopherol,^66^ α‐mangostin,^20^ spirulina,^67^ and resveratrol.^68^, ^69^ In the present study, we successfully developed electrospun PCL scaffolds as a means of delivering AA directly into the infarcted zone.

A tissue homogenizer was used to suspend fine AA particles within a HFIP solution before the addition of the PCL. AA is highly soluble in water, which is fully miscible with HFIP. However, previous electrospun mats produced using a HFIP/water solution were flimsy with large variations in fiber diameters. This instability in the electrospinning process is likely due to both the high surface tension and low evaporation rate of the water.^70^ Preparing suspension solutions allowed us to maintain the higher concentration of AA (0.3% wt/vol) and fabricate mats with uniform morphology. Our SEM images (Figure 2) display smooth, bead‐free fibers with random orientation for all scaffold groups. Cardiac ECM is composed of a fibrous, predominately collagen, matrix.^71^ Thus, morphologies similar to those presented in this study have been previously utilized for cardiac tissue engineering,^18^, ^29^, ^33^, ^72^ with the grafts supporting adhesion and survival of cardiac fibroblasts, coronary artery endothelial cells and mesenchymal stem cells.

Several works have highlighted the influence of electrospun PCL morphology and topology on the cellular response.^48^, ^73^, ^74^ Notably, the viability and gene expression of HUVECs are significantly influenced by the PCL fiber diameter.^50^ In the present study, the diameters were relatively uniform between groups, exhibiting a 3.7% difference in mean values, therefore it can be assumed that any differences obtained in vitro are a result of scaffold composition as opposed to scaffold morphology. While our SEM images are limited to visualization of the fibrous architecture, the adhesion of HUVECs may be improved with increased surface roughness at the nanoscale.^75^ Our group have previously modified the topography of PCL microfibers via solvent phase separation, demonstrating that the presence of small or large depressions significantly influence the proliferation and gene expression of a liver cell line.^49^ Therefore, future analyses into the effect of addition of AA on the fiber surface roughness and topology are warranted.

AA is an antioxidant vitamin with a mechanism of action of a one or two‐step oxidization reaction.^76^ As expected, addition of AA enhanced the antioxidant capabilities of the scaffolds (Figure 5a–c). DPPH is one of the most common methods for determining antioxidant activity and O_2_^•–^ and H_2_O_2_, as the direct products of oxygen reduction, are present within the post‐infarct.^77^ Our data indicates that both antioxidant scaffolds reduced the concentration of DPPH radicals with no difference between the groups, but only the higher concentration antioxidant scaffold significantly reduced the number of O_2_^•−^ radicals. Additionally, the scaffolds were incubated with media containing H_2_O_2_; note that results of the assay were normalized to account for the reduction in H_2_O_2_ concentration seen within the media only control, likely caused by the natural decomposition of the H_2_O_2_ into water and oxygen. The 0.3% AA scaffold significantly reduced the H_2_O_2_ concentration of over the course of a 6‐day period, suggesting that AA is bound within the hydrophobic PCL and protected from oxidation until release, via both degradation of and diffusion out of the fibers.

An increase in the AA content correlated with a slight but non‐significant increase in the hydrophilicity, and a significant increase in the ultimate tensile strength and the Young's modulus within the 0%–5% strain band (Table 2, Figure 3a). This has been reported in previous studies: encapsulating AA into polylactic acid scaffolds significantly increased their stiffness and strength,^47^ and a fibrous mat incorporating both AA and hyaluronic acid into PCL showed improved strength compared with the hyaluronic acid‐PCL scaffold.^45^ Similar trends have been observed when incorporating other bioactive molecules into PCL fibers, including proteins^32^ and decellularized ECM.^35^ Alipour et al. hypothesized that AA may function as an inactive filler in composite polymer scaffolds, increasing the resultant tensile strength.^78^

In addition, it is well established that increases in the degree of crystallinity increases the strength and stiffness of polymers. Our results show that addition of AA increased crystallinity of the scaffold, though no significance was observed (Table 3). A recent study investigated the influence of increasing AA concentrations on the crystallization kinetics of PCL films by preparing polymer and AA solutions and observing them dry in a dish.^79^ Interestingly, the crystallinity of the film was enhanced with addition of 1 and 2 wt/vol% AA into the polymer solution, but decreased when the concentration was increased further to 5 wt/vol%. This correlates with our results, with the 0.15% AA scaffold displaying the largest degree of crystallinity, while the crystallinity of the 0.3% AA scaffold was still greater than the PCL control. Eesaee et al. concluded that the lower concentrations of AA increased the nucleation density of the PCL, while higher concentrations resulted in aggregates of ascorbate which impeded mobility of the PCL chains during crystallization. It is possible that a similar phenomenon occurred during electrospinning. Although the concentrations of AA used in this study were almost tenfold lower, the rate of crystallization during electrospinning is significantly faster due to the fine diameter and large surface area of the polymer jet, which significantly enhances the evaporation rate of the solvent. Likewise, we observed that the fabrication process influenced the melting points and crystallinity of all scaffolds, which all decreased after common thermal treatment. This is expected, as electrospinning parameters such as the distance between the needle tip and the mandrel and the rate of solvent evaporation have all been shown to influence the resultant polymer crystallinity.^60^

Previous studies encapsulating AA within electrospun fibers have shown no cytotoxic effects against human dermal fibroblasts^46^, ^47^ and H9C2 rat myoblasts.^13^ Similarly, a polycaprolactone‐based polyurethane incorporating AA as the chain extender has shown promising protective benefits against cardiovascular cell lines exposed to oxidative stress conditions.^18^, ^19^, ^80^, ^81^ Endothelial cells have a range of functions within the cardiac system including regulating blood flow, cardiomyocyte growth, contractility, apoptosis and the inflammatory response following ischemia.^82^ Endothelial dysfunction plays a role in the pathogenesis of atherosclerosis; subsequently, most patients with myocardial infarction show signs of persistent endothelial dysfunction.^83^, ^84^ ROS are known to contribute to endothelial dysfunction by reducing the bioavailability of nitric oxide, ultimately disrupting vasoreactivity and signaling between endothelial cells and cardiomyocytes.^82^ Supplementation with ascorbate has been shown to improve endothelial tone and function.^85^ In the present study, all scaffold groups maintained culture of HUVECs for up to 6 days (Figure 6a,b), indicating no cytotoxicity in vitro. Although the dsDNA results indicate that no real cell proliferation was observed, the suggested application for the scaffold is the reduction to oxidative stress within the infarcted microenvironment, thereby improving the survival and functionality of the implanted cells. Additional in vitro analyses using cardiovascular cell lines under a simulated hypoxic environment are warranted and would provide further information on the cardioprotective effects of the antioxidant scaffold.

This study is not without its limitations. Firstly, additional analyses are required to determine the total amount of AA present within the fibers and the release kinetics of the AA in vitro. In the present study, Fourier‐transform infrared analyses were conducted using a Nicolet™ iS™10 Spectrometer (ThermoFisher Scientific, United Kingdom) but results were inconclusive (data not shown). The results of our radical scavenging assays and the increase in tensile properties indicates the presence of AA within our fibers, but the total concentration is below that of the limit of detection of our instrument. The resultant concentrations of AA in the mats were 1.5% wt/wt and 3% wt/wt respective to the mass of PCL. These calculations assume electrospinning of a homogenous solution, thereby limited aggregation of AA particulates, and no degradation of the AA during the solution preparation and scaffold fabrication stages. It is therefore probable that the overall quantity of AA present in the fibers is lower than our estimated values.

Furthermore, within the DPPH and O_2_^•–^ scavenging assays the scaffolds were incubated for 30 and 5 min, respectively, and displayed immediate antioxidant activity. In comparison, it took 3 days until a significant reduction in the H_2_O_2_ concentration was detected. The notable difference between these assays is the sterilization and washing stages undertaken prior to scaffold incubation with the H_2_O_2_‐doped media. Due to its high solubility in water, it is likely that the AA on the surface of the fibers was removed by the ethanol and PBS―this burst release is typically seen by AA out of fibrous mats.^25^, ^47^, ^86^ To avoid release of AA during the washing stages, other available sterilization techniques should be considered. A suggestion is using oxygen plasma treatment, which would clean the scaffold without altering the bulk properties of the material and may retain more of the antioxidant capabilities.^49^ Similarly, other methods of incorporating AA into biocompatible polymers may be more suitable. Other previously published methods include chemical modification of polyurethanes^80^, ^87^, ^88^ and surface modification via plasma‐assisted grafting.^89^, ^90^ With relevance to the present study, Zhao et al. compared the mechanical properties and release rates of PCL scaffolds containing an AA derivative fabricated via emulsion, coaxial, and blend electrospinning.^25^ In their study the coaxial fibers performed poorly, releasing 100% of the antioxidant within 24 h. Yet, release rates of AA and its derivatives encapsulated within the core of coaxial fibers within literature are varied. Cumulative release of magnesium l‐AA 2 phosphate only reached 40% after 7 days within PCL and gelatin fibers,^13^ while blended chitosan and poly(vinyl alcohol) fibers released 74% of AA within 30 h.^86^ This highlights the flexibility of electrospun fibers in tailoring release rate, depending on the fiber morphology and composition, material or material blends used, and overall concentrations of drugs loaded within the fibers.

The degradation kinetics of PCL are well reported in literature, with PCL grafts exhibiting a slow degradation rate under normal conditions compared to other synthetic polymers (around 2–3 years^91^, ^92^). This makes PCL a suitable material for long‐term drug release, but potentially hinders its use as a regenerative therapy following myocardial infarction, since research proposes that the ideal cardiac scaffold degrades within 2 months.^93^ It is expected that our AA fibers would degrade at a faster rate than the pure PCL fibers due to the slight increases in crystallinity and surface wettability, as has been previously reported.^45^, ^79^ Additionally, the mechanical properties of a tissue‐engineered graft should ideally match that of the native tissue^94^ as material stiffness has been shown to influence cell behavior.^95^, ^96^ Infarcted cardiac tissue can be up to 1000% stiffer than its healthy counterpart (10–100 kPa),^97^, ^98^ thus the mechanical properties of our cell‐free therapeutic patches are approaching that of infarcted tissue. It is important to note that a single collagen fibril exhibits very high stiffness, in the range of 5–7.5 GPa,^99^ far greater than that of electrospun fibrous mats. Since no single material perfectly captures the biological and mechanical properties of the native tissue, polymers are often combined during synthesis (producing copolymers) or fabrication (producing blended scaffolds), permitting fine tuning of the resultant properties and overcoming the drawbacks of a single material. As such, cardiac scaffolds prepared with PCL are commonly mixed with or layered alongside natural polymers, particularly gelatin,^13^, ^33^, ^100^, ^101^ collagen,^102^, ^103^ chitosan,^104^ or combinations of these.^103^, ^105^ Within these systems, PCL provides the mechanical strength and stiffness and prolonged degradation kinetics that is lacking in the natural polymers, enhancing handleability and functionality as a left ventricle graft, while the natural polymers enhance the resultant hydrophilicity and biocompatibility. This provides researchers with an additional means of altering the release rates of loaded molecules, not only due to the aforementioned properties, but also fundamental changes in the material microstructure and resultant surface area available for diffusion.

Finally, AA is well known to promote collagen deposition in cardiovascular cells, including fibroblasts^46^, ^47^ and smooth muscle cells.^106^, ^107^ This may be advantageous: collagen is a major component of the cardiac ECM and following myocardial infarction there is a dramatic increase in the activity of matrix metalloproteinases (MMPs) which degrade the ECM.^108^ In the initial healing stages, activation of fibroblasts to myofibroblasts occurs which deposit scar tissue to prevent rupture of the left ventricle. However, these changes in ECM composition contribute toward ventricular remodeling resulting in impaired cardiac function. Since ROS also play a role in the activation of MMPs and deposition of collagen,^109^ it may be that the biological response to an implanted AA releasing scaffold will be different depending on the length of time following myocardial infarction. While it is probable that AA will promote regeneration and repair of the remodeled heart, further investigation is required to investigate the implications of AA on collagen deposition within, and potential stiffening of, the infarcted region.

The results in this study serve as proof of concept for the use of blended PCL/AA fibers for reducing oxidative stress within the infarcted cardiac microenvironment. However, additional in vitro analyses such as gene expression, quantification of intracellular oxidative stress and angiogenic assays using other cardiac cell lines cultured under normoxic and hypoxic microenvironments is recommended to demonstrate the safety and efficacy of the scaffold for treatment of myocardial infarction.

## 5 CONCLUSION

In this study, we developed an implantable antioxidant scaffold via electrospinning techniques, encapsulating AA in PCL microfibers. Our results indicate that the incorporation of AA improved the mechanical properties of the fibers without altering the surface morphology or wettability. Furthermore, the scaffold's antioxidant capability significantly improved as measured by DPPH, superoxide and hydrogen peroxide radical scavenging assays. The viability of HUVECs cultured on the scaffold showed no difference between scaffold groups, indicating that incorporation of the AA had no cytotoxic effects in vitro. Further optimization and investigation of this system is recommended for cardiac tissue engineering applications.

## AUTHOR CONTRIBUTIONS

**Ella‐Louise Handley:** Conceptualization (equal); data curation (lead); formal analysis (lead); funding acquisition (equal); investigation (lead); methodology (lead); writing – original draft (lead); writing – review and editing (lead). **Anthony Callanan:** Conceptualization (equal); funding acquisition (equal); supervision (lead); writing – review and editing (supporting).

## Data Availability

The data that support the findings of this study are available from the corresponding author upon reasonable request.
